# An unusual presentation of multiple myeloma: a case report

**DOI:** 10.1186/1752-1947-1-84

**Published:** 2007-09-10

**Authors:** Catherine B Molloy, Rahul A Peck, Stephen J Bonny, Simon N Jowitt, John Denton, Anthony J Freemont, Abbas A Ismail

**Affiliations:** 1 Rheumatology, St. Michaels Hospital, Toronto, Canada; 2 Rheumatology, Stockport NHS Trust, UK; 3 Haematology, Stockport NHS Trust, UK; 4 Haematology, Salford Royal NHS Trust, UK; 5 Osteoarticular Pathology, Manchester University, UK

## Abstract

Multiple myeloma can occasionally manifest with joint disease. We report the case of an individual with a progressive bilateral carpal syndrome and a symmetrical severe seronegative polyarthritis and joint swelling. Investigations revealed an erosive seronegative inflammatory arthritis in association with bilateral carpal tunnel syndrome, anaemia, hepatic impairment and nephrotic-range proteinuria. Synovial fluid cytology demonstrated plasmablasts and multinucleated cells with products of chondrolysis. The diagnosis of multiple myeloma (with secondary amyloidosis) was made on serum protein electrophoresis and bone marrow biopsy.

The relationship between myeloma and joint disease is discussed, highlighted by the presence in this case of all three pathogenic features associated with arthritis in myeloma patients- an erosive arthritis, carpal tunnel syndrome and an invasive tumoural arthritis.

## Background

Multiple myeloma is a malignant proliferation of plasma cells producing a monoclonal paraprotein. Multiple myeloma can present in a range of ways, for example, hypercalcaemia, hyperviscosity, renal failure and bone pains/fractures. We report an unusual presentation of multiple myeloma in the form of symmetrical severe polyarthritis and joint swelling.

## Case presentation

A 55 year old lady referred to the rheumatology clinic with a 3 month history of progressive disabling polyarthralgia and joint swelling, a 5 kg weight loss and fatigue. The predominant joints affected were her knees, shoulders, wrists and small hand joints; her hand function was so impaired at the time of presentation that she was no longer able to feed herself. She denied joint stiffness, thigh pain, a history of skin rash, gastrointestinal or genitourinary symptoms.

On examination she was pale and cachectic. She had generalised soft tissue swelling of her hands, with markedly reduced wrist movements, but without synovitis. Tinel's and Phalen's tests were strongly positive bilaterally consistent with carpal tunnel syndrome. Moderate cool effusions were present in both knees. No synovitis was present elsewhere and the rest of her systemic examination was normal.

She had a normochromic anaemia with a borderline leucopaenia (Hb 65 g/l, MCV 80 fl, WCC 3.9 × 10^9^/l, platelets 200 × 10^9^/l) and a grossly raised ESR (>140 mm/hr). She was hypercalcaemic (corrected calcium 3.15 mmol/l, phosphate 1.82 mmol/l, alkaline phosphatase 102 U/l) with deranged liver function (LDH 1085 U/l, AST 46 U/l, normal bilirubin, albumin and globulin levels). Significant renal disease was evident (urea 22 mmol/l, creatinine 407 μmol/l), +1 of blood and protein on urinalysis, a creatinine clearance of 16 ml/min and nephrotic range proteinuria (5.29 g/d). Hand radiographs showed wrist joint space narrowing with juxta-articular erosions.

**Figure 1:**
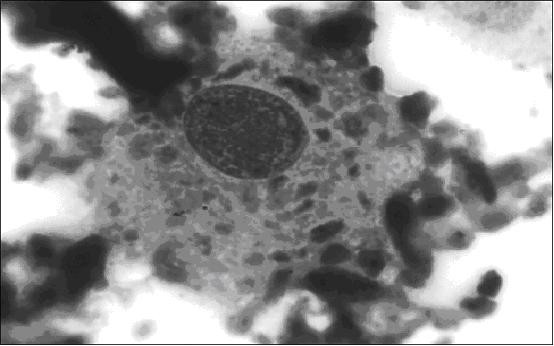
Knee synovial fluid: plasmablast-like cell containing particles of phagocytosed degenerate articular cartilage surrounded by suspended degenerate cartilage (Jenner Giemsa, ×1000). Informed consent was given for publication from the patient's next-of-kin.

Left knee synovial fluid cytology revealed atypical cells resembling plasmablasts and multinucleate cells, as well as changes consistent with chondrolysis, figure 1. It was felt this was due to malignant infiltration of cartilage, with bone and cartilage degradation products present in the fluid. Wrist aspiration was dry.

Subsequently, rheumatoid factor, ANA, ENA and ANCA were all negative and a non-contrast CT scan of her thorax, abdomen and pelvis did not identify any abnormalities of the viscera or the skeleton.

A panhypogammaglobulinaemia was identified [IgG was 3.7 g/l (8–16), IgA and IgM were both 0.1 g/l (1.4–4, 0.5–2)]. Electrophoresis identified a small paraprotein band (2 g/l), and a large amount of free kappa light chains in both the serum and the urine (8.8 mg/l). Haematological advice was sought and bone marrow biopsies were undertaken, demonstrating a heavy (>90%) infiltration by plasma cells including atypical forms, with a marked reduction in granulopoiesis and erythropoiesis. Amyloid protein was also identified in the walls of blood vessels within the trephine biopsy.

Thus a diagnosis of aggressive multiple myeloma was made (stage IIIB) and the patient was treated with aggressive VCADVCAD chemotherapy (vincristine, cyclophosphamide, adriamycin and dexamethasone). Unfortunately, she died from pneumonia seven weeks after presentation.

## Discussion

We have described the initial presentation of an aggressive multiple myeloma with an erosive seronegative polyarthritis due to direct myelomatous joint infiltration. On review of the literature, a few case reports have described articular presentations of the plasma cell dyscrasias-multiple myeloma (MM) [1,2], monoclonal gammopathy of uncertain significance (MGUS) [1,2] and Waldenström's macroglobulinaemia [3].

Joint involvement in myeloma is typically an oligoarthritis [1] or a polyarticular rheumatoid-like pattern, as seen in this case. Though individuals with myeloma are at greater risk of both septic arthritis and gouty arthritis [3], other pathophysiological mechanisms have been postulated to account for joint disease. Firstly, local synovial precipitation of cryoprecipitable paraproteins [1,4] or immunoglobulin crystals [4] may activate the inflammatory response resulting in an erosive arthritis [2]. Secondly, a carpal tunnel syndrome may develop from intrasynovial deposition of amyloid protein or immunoglobulins [5]. Finally, juxta-articular plasmacytic lesions may infiltrate the synovium and synovial fluid resulting in a 'tumoural arthritis'. This direct tumour invasion of the joint has been identified in other primary haematological malignancies [3,6-8], however it is an extremely rare manifestation of the plasma cell dyscrasias, having only previously been described in 2 individuals with myeloma [3,8]. This case demonstrated all of these three pathogenic features- an erosive arthritis, carpal tunnel syndrome and an invasive tumoural arthritis.

This case is also unique in that the synovial fluid analysis yielded the ultimate diagnosis. In a case series of 9 individuals with a monoclonal gammopathy (MGUS or MM) and arthritis, the majority [5] were diagnosed with the plasma dyscrasia first, synchronous diagnoses were made in 3, and arthritis was the presenting feature in only 1 case [1]. There is no information on the prognosis of cases presenting in this manner, but based on the presence of anaemia, hypercalcaemia, renal impairment, advanced lytic bone lesions and high tissue M-component levels in this case, a high myeloma tissue mass was present, related to a poor prognosis [9].

## Conclusion

We report the case of a patient presenting with tumoural arthritis and carpal tunnel syndrome from an aggressive myeloma. This case stresses the importance of analysing the synovial fluid of any patient with an atypical joint disease or a suspected plasma cell dyscrasia for cytology and immunohistochemistry, micro-organisms, crystals, and also for immunoglobulins and amyloid.

## Competing interests

The author(s) declare that they have no competing interests.

## Authors' contributions

The authors were involved in the writing of the manuscript or patient clinical care. All authors read and approved the final manuscript.
